# Asymptomatic Renal Colonization of Humans in the Peruvian Amazon by *Leptospira*

**DOI:** 10.1371/journal.pntd.0000612

**Published:** 2010-02-23

**Authors:** Christian A. Ganoza, Michael A. Matthias, Mayuko Saito, Manuel Cespedes, Eduardo Gotuzzo, Joseph M. Vinetz

**Affiliations:** 1 Instituto de Medicina Tropical Alexander von Humboldt, Universidad Peruana Cayetano Heredia, Lima, Peru; 2 Division of Infectious Diseases, Department of Medicine, University of California San Diego School of Medicine, La Jolla, California, United States of America; 3 Department of International Health, Johns Hopkins Bloomberg School of Public Health, Baltimore, Maryland, United States of America; 4 Department of Microbiology, Universidad Peruana Cayetano Heredia, Lima, Peru; 5 Asociacion Benefica PRISMA, Lima, Peru; 6 Instituto Nacional de Salud, Lima, Peru; Cambridge University, United Kingdom

## Abstract

**Background:**

Renal carriage and shedding of leptospires is characteristic of carrier or maintenance animal hosts. Sporadic reports indicate that after infection, humans may excrete leptospires for extended periods. We hypothesized that, like mammalian reservoir hosts, humans develop asymptomatic leptospiruria in settings of high disease transmission such as the Peruvian Amazon.

**Methodology/Principal Findings:**

Using a cross-sectional study design, we used a combination of epidemiological data, serology and molecular detection of the leptospiral 16S rRNA gene to identify asymptomatic urinary shedders of *Leptospira*. Approximately one-third of the 314 asymptomatic participants had circulating anti-leptospiral antibodies. Among enrolled participants, 189/314 (59%) had evidence of recent infection (microscopic agglutination test (MAT0 ≥1∶800 or ELISA IgM-positive or both). The proportion of MAT-positive and high MAT-titer (≥1∶800) persons was higher in men than women (*p* = 0.006). Among these people, 13/314 (4.1%) had *Leptospira* DNA-positive urine samples. Of these, the 16S rRNA gene from 10 samples was able to be sequenced. The urine-derived species clustered within both pathogenic (n = 6) and intermediate clades of *Leptospira* (n = 4). All of the thirteen participants with leptospiral DNA in urine were women. The median age of the DNA-positive group was older compared to the negative group (*p*≤0.05). A group of asymptomatic participants (“long-term asymptomatic individuals,” 102/341 (32.5%) of enrolled individuals) without serological evidence of recent infection was identified; within this group, 6/102 (5.9%) excreted pathogenic and intermediate-pathogenic *Leptospira* (75–229 bacteria/mL of urine).

**Conclusions/Significance:**

Asymptomatic renal colonization of leptospires in a region of high disease transmission is common, including among people without serological or clinical evidence of recent infection. Both pathogenic and intermediate *Leptospira* can persist as renal colonization in humans. The pathogenic significance of this finding remains to be explored but is of fundamental biological significance.

## Author Summary

Leptospirosis is a bacterial disease commonly transmitted from animals to humans. The more than 200 types of spiral-shaped bacteria (spirochetes) in the genus *Leptospira* are classified as pathogenic, intermediately pathogenic, or saprophytic (meaning not causing infection in any mammal) based on their ability to cause disease and on genetic information. Unique among the spirochetes that infect humans, *Leptospira* live both in the environment (in surface waters and moist soils), and in mammals, where they cause chronic infection by colonizing kidney tubules. Infected animals are the source of human infection, but humans have not been systematically studied as chronic *Leptospira* carriers. In our study, we found that more than 5% of people (in fact, only women) in a rural Amazonian village, without clinical evidence of infection by *Leptospira*, were chronically colonized by the bacteria. Chronic infection was not associated with a detectable immune response against the spirochete. Pathogenic and intermediately pathogenic *Leptospira* caused asymptomatic, chronic kidney infections. Future work is needed to determine whether such chronic infection can lead to human-to-human transmission of leptospirosis, and whether subtle measures of kidney disease are associated with asymptomatic, long-term leptospiral infection.

## Introduction

Leptospirosis is a zoonotic disease caused by spirochetes of the genus *Leptospira*. Found worldwide, leptospirosis is more common in tropical and sub-tropical areas where environmental and socioeconomic conditions favor its transmission. It has been identified in recent years as a global public health problem because of its increased mortality and morbidity. The disease is principally transmitted to humans indirectly by contact with water or soil contaminated with the urine of domestic and wild animals with persistent renal infection by *Leptospira* [1]–[3].

The tropical climate of the Peruvian Amazon region of Iquitos is ideal for the maintenance and transmission of leptospirosis. In developing countries, impoverished populations typically live either in rural areas or under highly crowded conditions in urban slums. These factors increase the risk of human exposure to the urine of *Leptospira*-infected animals [4],[5]. In the Iquitos region, leptospirosis is common. Seropositivity as seen in cross-sectional surveys is high [6]; more than half of patients presenting to urban and rural community-based health posts with non-malarial acute febrile illness have been observed to have diagnostic levels of anti-leptospiral antibodies suggestive of acute leptospirosis [4]. The majority of patients enrolled presented with a self-resolving undifferentiated febrile illness with 70% of them having antibodies against a newly described *Leptospira* species, *L. licerasiae* [7]. These data suggest that exposure to *Leptospira* is common in daily life in this tropical setting [5],[8], and that, in general, Iquitos is accurately classified as hyper-endemic for leptospirosis infection.

Leptospirosis in humans is frequently misidentified because of several factors: 1) variable and nonspecific clinical presentation; 2) lack of awareness of the disease among clinicians; and 3) difficulty in access to reliable and rapid diagnostic tests. Clinical manifestations, when present, vary from a mild ‘flu-like’ febrile illness to a severe disease variably including jaundice, renal failure, pulmonary hemorrhage, refractory shock and other grave manifestations. However, many if not most people infected by *Leptospira* develop sub-clinical disease or have very mild symptoms, and thus do not seek medical attention [1],[2]. Asymptomatic infection, common in endemic areas, has been reported in several studies [8]–[12]. For example, in one study, 9–48% of healthy subjects were diagnosed as having asymptomatic leptospiral infection by serology (ELISA-IgM) and PCR [10]. However, in this study, the identity of the infecting strains could not be determined because of study design. We have observed in one study that patients can have asymptomatic leptospiruria for prolonged periods of time [4]. Hence an essential question about the pathogenicity of *Leptospira* remains: are some serovars are more likely than others to establish asymptomatic renal infection in man?

Renal colonization and persistent shedding of leptospires is characteristic of carrier or maintenance animal hosts [13]–[15]. Animals, especially rodents, are known reservoirs of pathogenic *Leptospira* species, but rarely develop symptoms and are not impaired by the infection of their kidneys. After infection, humans can also excrete leptospires into the urine transiently for weeks or, more rarely, months or more [1],[2],[16].

We hypothesized that like mammalian reservoir hosts, humans develop asymptomatic leptospiruria, including pathogenic *Leptospira* such as *L. interrogans* and intermediate pathogens such as the newly discovered *L. licerasiae* [7]. To test this hypothesis, we carried out a cross-sectional, population-based study in a rural village near the city of Iquitos to identify the presence and species of infecting *Leptospira* directly in the urine of healthy ambulatory people. If found, we reasoned that the high prevalence of asymptomatic urinary infection might provide fundamental insights into the nature of *Leptospira*-human interactions, where humans are considered to be accidental hosts. Such a finding would also provide the basis for understanding mechanisms of naturally acquired immunity in human leptospiral infection.

## Materials and Methods

### Ethics Statement

This study was approved by the Human Subjects Protection Program, University of California San Diego, and the Ethical Committees of Asociacion Benefica PRISMA, Lima, Peru, and Universidad Peruana Cayetano Heredia, Lima, Peru. All human subjects provided written informed consent before being enrolled in the study.

### Description of Study Area

This study was carried out in the village of Padrecocha, a rural community near Iquitos, located north of the city along the Nanay River, a tributary that branches from the Amazon River 15 km downstream from Iquitos. The climate is tropical: rainfall averages 300 mm per year and temperatures range from 21.8°C to 31.6°C; the village is surrounded by a vast expanse of humid tropical rainforest. The population of this village is approximately 1,500. Most inhabitants live in brick houses, and their water supply comes from wells and local streams. These water sources harbor pathogenic and intermediate-pathogenic *Leptospira* [5]. Residents use water from wells or from the local streams for their daily needs (cooking, bathing and washing clothes). There is no sewage system; most households have pit latrines. Livestock (mostly chickens, pigs, and cattle) roam free through the village and its streams; the inhabitants observe rats frequently.

### Enrollment

**Figure 1:**
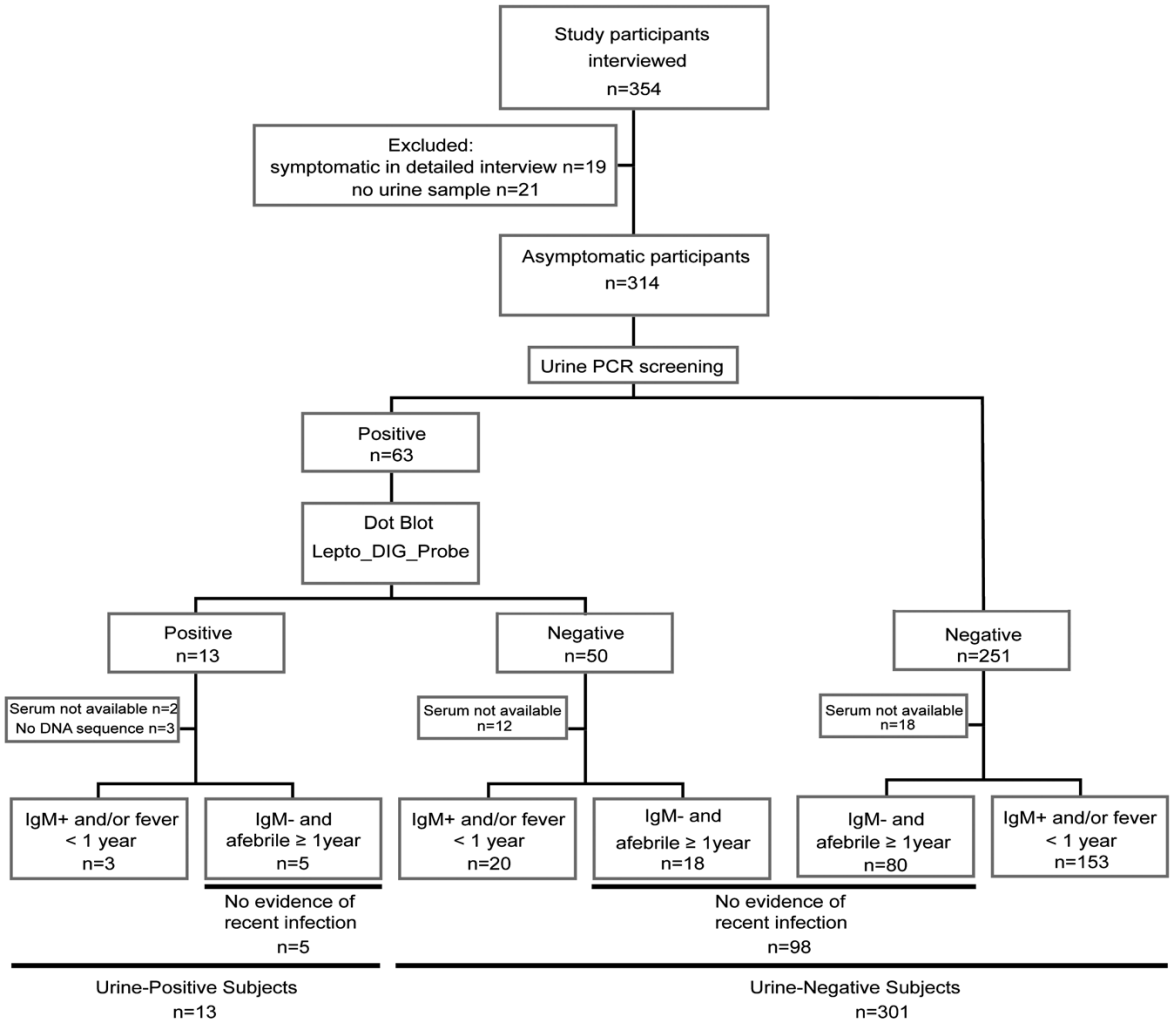
Flow chart showing study subject enrollment and sample processing.

Using a whole-village canvassing strategy to develop a set of candidate houses from which to randomly select asymptomatic inhabitants of the rural village Padrecocha of age ≥5 years for enrollment. Subjects were excluded if they had fever within the previous 2 weeks or if they declined participation (Figure 1). All participants were clinically evaluated and subjected to an epidemiologic questionnaire. Whole blood (5 mL) and urine samples (5—50 mL) were collected from each enrollee.

### Serological Testing

Venous blood samples were drawn into tubes without anticoagulant (Becton-Dickinson, USA) and transported to the study laboratory within 4 hr at ambient temperature. Serum was separated, frozen in 1 mL aliquots at −20°C, and transported on dry ice to the National Leptospirosis Reference Laboratory at the Instituto Nacional de Salud (INS) in Lima, where the presence of anti-leptospiral antibodies was determined. An ELISA incorporating 6 pathogenic serovars (strains)–Icterohaemorrhagiae (RGA), Australis (Ballico), Bratislava (Jez Bratislava), Ballum (MUS127), Canicola (Hond Utretch IV), Cynopteri (3522 C), and Grippotyphosa (Moskva V)–was used to detect anti-leptospiral IgM antibodies. An ELISA IgM result of 11.0 IU/mL or more was considered to be positive [4],[7]. Microscopic agglutination testing (MAT) was performed using 25 leptospiral antigens, using the Centers for Disease Control and Prevention (CDC) panel [17]. MAT titers were reported as the reciprocal of the number of dilutions still agglutinating 50% of live bacterial antigen and a titer of 1∶100 or more was considered as positive.

### Detection of Leptospiral DNA in Urine Samples

#### Sample collection and DNA extraction

Urine samples were collected in 50 mL sterile polypropylene centrifuge tubes and centrifuged at 3,000 g for 30 min at room temperature. DNA from the pellet was extracted using the QIAamp® DNA Mini Kit (Qiagen, USA) following manufacturer's directions. DNA was extracted from samples on-site in the Iquitos laboratory the same day of collection and stored at −20°C.

#### Quantitiative real time PCR (qPCR) screening

All urine samples collected were screened for the presence of pathogenic and intermediate-pathogenic *Leptospira* using a published qPCR TaqMan assay targeting the leptospiral 16S ribosomal gene [18]; this was performed on-site in our Iquitos laboratory using an Opticon 2 real-time PCR machine (MJ Research, USA). The assay protocol was modified from the published version by using the fluorescent probe at a final concentration of 0.2 *µ*M, primers at a final concentration of 0.5 *µ*M, and a 20 *µ*L reaction volume [5]. Standard curves for quantification were made using *Leptospira interrogans* serovar Copenhageni strain M20. Standards were prepared as follows. Leptospires were counted using a Petroff-Hauser counting chamber (Hauser Scientific, USA) and serially diluted with sterile double-distilled H_2_O to 10^8^ to 10^0^ leptospires/ml. Genomic DNA was subsequently prepared using the DNeasy Tissue Kit (Qiagen, USA). Standards were run in triplicate to generate a standard curve with each run. A negative result was assigned where no amplification occurred before 40 cycles. Controls lacking template were extracted and added to qPCR master mix to detect the presence of contaminating DNA.

#### Nested PCR amplification of leptospiral 16S rDNA gene and sequencing of PCR products

To address potential limitations of sensitivity, a nested PCR strategy was used using the general eubacterial outer primers fD1 and rD1 [19] in a first round of amplification followed by amplification with the specific leptospiral 16S rDNA primers lepto16S11f and lepto16S1338r [5]. Total genomic DNA from 63 qPCR-positive urine samples was amplified using the universal bacterial 16S rDNA primers fD1/rD1 as described previously. PCR products were purified from 1.0% agarose gels in TAE buffer using the QIAquick Gel Extraction Kit (Qiagen, USA) according to manufacturer's directions, diluted 1∶100 in sterile double-distilled water, subjected to a second round of amplification using the nested primers lepto16S11f and lepto16S1338r with a previously described protocol [5], and then cloned into the pCR2.1-TOPO vector (Invitrogen, USA). Recombinant plasmids containing nested PCR products were then transformed into TOP10 cells (Invitrogen, USA) and plated on LB agar containing 100 *µ*g/mL ampicillin. Individual clones were grown overnight in LB broth containing 100 *µ*g/mL ampicillin. Plasmid DNA from these clones was purified using the QIAprep Spin Miniprep Kit (Qiagen, USA) and cycle sequenced. Sequencing was performed on an ABI 3100 automated sequencer (Applied Biosystems, USA) using the M13 forward and reverse primers (Invitrogen, USA). Reaction conditions were according to manufacturer's directions.

#### Southern hybridization (dot blot)

**Figure 2:**
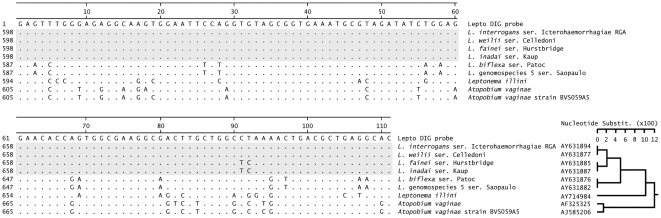
Sequence alignment of the *Leptospira* 16 s rRNA gene probe and target. The digoxigenin-labeled *Leptospira* 16 s rRNA gene probe (111 bp) aligned with fragments of the 16 s rRNA genes of *Leptospira* (n = 6), *Leptonema* (n = 1) and *Atopobium* (n = 2) species. Pathogenic and intermediate *Leptospira* species are shaded in gray.

After the initial evaluation of the DNA sequences of the first PCR-positive samples we observed a high rate of false positivity for the nested PCR in urine samples (75%, data not shown). The 16S ribosomal RNA gene sequences of the organisms present in the urine of the false positive PCR samples were frequently identical to the 16S ribosomal RNA gene sequence of the bacterium *Atopobium vaginae*, an organism that has been recently recognized in the microflora associated with bacterial vaginosis [20]. The 16S ribosomal RNA gene inner nested primers used to amplify *Leptospira* shared sufficient sequence homology with the *Atopobium* 16S rDNA gene that under the conditions described these primers generated amplicons for both *Leptospira* (∼1327 bp) and the *Atopobium* (∼1345 bp) species, making it difficult to distinguish this amplicon by agarose gel electrophoresis alone. To overcome this problem we developed a dot blot hybridization assay using a digoxigenin (DIG)-labeled DNA probe. Briefly, a 111bp DIG-labeled DNA probe (Lepto_DIG_probe) was synthesized by PCR-labeling with the PCR DIG Probe Synthesis Kit (Roche, USA). *Leptospira interrogans* serovar Copenhageni strain Fiocruz L1-130 genomic DNA was used as template; primers lepto16S620f (5′-GAGTTTGGGAGAGGCAAGTGGAATTC-3′) and lepto16S730r (5′-GTGCCTCAGCGTCAGTTTTAGGCC-3′) for the probe synthesis. These primers were selected from conserved regions of the 16S rDNA genes of pathogenic and intermediate-pathogenic leptospiral species, but were absent from the saprophytes (Figure 2). Published 16S ribosomal RNA sequences (n = 178) retrieved from the Ribosomal Database Project II release 9 (http://rdp.cme.msu.edu) and aligned using clustalW 1.83 (http://www.ebi.ac.uk/clustalw) were used. The PCR reaction mix was prepared following the manufacturer's directions with the primers at a final concentration of 0.4 *µ*M and a 1∶12 ratio of DIG-dUTP:dTTP nucleotides. The synthesis was conducted in a DNA Engine PCT-200 Peltier Thermal Cycler (MJ Research). The amplification protocol consisted of 95°C for 2 min, followed by 30 cycles of amplification, each cycle consisting of 95°C for 10 s, 64°C for 30 s, and 72°C for 2 min.

#### Dot blotting of DNA samples

Genomic DNA from urine samples and controls were PCR-amplified using the aforementioned nested PCR assay, and the PCR products were separated by agar electrophoresis and purified from the agarose gels. PCR products were then denatured by boiling for 10 min in a denaturing solution (0.4 M NaOH/10 mM EDTA, final concentration), then cooled on ice and spotted into an Immobilon-Ny+ Charged Nylon Transfer Membrane (Millipore, USA) using a 96-well vacuum manifold (Bio-Dot Microfiltration Apparatus, Bio-Rad, USA). After spotting the samples, the membrane was air-dried and the DNA was fixed by UV cross-linking using the Stratalinker UV Crosslinker 2400 (Stratagene, USA). The spotted membrane was hybridized overnight at 56°C with 5 pmol of Lepto_DNA_probe in 3.5 mL of DIG Easy Hyb hybridization buffer (Roche, USA) according to manufacturer's directions. After hybridization and stringency washes, the blot was developed using a chemiluminescent assay with Anti-Digoxigenin-AP and CPD-Star (Roche, USA) according to manufacturer's directions. For detection of the chemiluminescent signal, X-ray film was exposed to the blot for 1 minute.

#### Colony hybridization assay

The dot-blot positive samples contained a high proportion of contaminating *Atopobium* DNA (data not shown). Thus to efficiently distinguish clones containing leptospiral 16S rDNA amplicons for sequencing, we developed a colony hybridization assay using the Lepto_DIG_probe whereby we could screen all the transformants on a plate simultaneously. Briefly, 16S ribosomal RNA genes previously PCR-amplified using the nested PCR assay from dot blot-positive samples (n = 13) were cloned using the PCR2.1-TOPO vector in *E. coli* as previously described. Transformed bacteria were grown at 37°C in Petri dishes (LB agar with 100 *µ*g/mL ampicillin) overnight. Colonies bearing the cloned 16S rDNA gene were adsorbed onto positively charged circular nylon membranes (Immobilon-Ny+ membrane discs, Millipore) and lifted from the agar plate. After adsorption, the bacteria were denatured in the membranes and their DNA fixed to them by UV cross-linking. A mean of 4 Petri dishes (or ∼200 colonies) with transformed bacteria bearing the 16S ribosomal RNA gene per urine sample were screened with this method. Membranes were hybridized with the Lepto_DIG_probe and developed as described above. Positive colonies in this assay were handpicked and grown overnight. Plasmid DNA was purified and cycle sequenced as described previously to identify the present leptospires to the species level.

#### Identification of infecting *Leptospira* by phylogenetic analysis of 16S rRNA gene sequences from urine samples

DNA sequences were assembled (using 4- to 6-times coverage per nucleotide base) using the program CAP3 (http://pbil.univ-lyon1.fr/cap3.php) then aligned using CLUSTALW v1.83 with default parameters. *Leptospira* 16S ribosomal RNA gene sequences from GenBank were used as controls in the tree (Table 1). Sequences derived from urine samples were analyzed simultaneously and with identical parameters for the nucleotide substitution model. Missing (gaps) and ambiguous characters were excluded from the analysis. However, a separate data partition was included whereby gaps were coded as binary state data; with gap characters coded as 1 while all others were coded as 0. This data partition was analyzed using the restriction site model as implemented in MrBayes. A phylogenetic dendrogram was generated using MrBayes v3.1.2 for Macintosh (http://mrbayes.csit.fsu.edu/) running for0 3,000,000 generations. The data were analyzed using the GTR+I+G nucleotide substitution model with gamma-distributed rates and proportion of invariant sites. The resulting data sets were then analyzed using flat priors for the substitution rate parameters [5],[7]. New leptospiral 16S sequences were deposited into GenBank (Table 1).

**Table 1:** GenBank accession numbers of leptospiral 16S rRNA gene sequences used in this study to construct phylogenetic trees, including new sequences from patient urine samples.

Accession No.	Strain designation or source
*Leptonema*	
AY714984	*Leptonema illini* serovar Illini strain 3055
Pathogenic *Leptospira*	
AY631877	*Leptospira weilii* serovar Celledoni strain Celledoni
AY631880	*L. alexanderi* serovar Manhao 3 strain L 60
AY631881	*L.* genomosp. 1 serovar Sichuan
AY631883	*L. santarosai* serovar Shermani strain LT 821
AY631884	*L. borgpetersenii* serovar Ballum strain Mus 127
AY631886	*L. noguchii* serovar Panama strain CZ 214
AY631894	*L. interrogans* serovar Icterohaemorrhagiae strain RGA
AY631895	*L. kirschneri* serovar Cynopteri strain 3522
FJ154542	*L. interrogans* serovar Copenhageni strain M 20
Intermediately Pathogenic *Leptospira*	
AY631885	*L. fainei* serovar Hurstbridge strain BUT 6
AY631887	*L. inadai* serovar Kaup strain LT 64–68
AY631891	*L. inadai* serovar Aguaruna strain MW 4
AY631896	*L. inadai* serovar Lyme strain 10
AY792329	*L. broomii* strain L065
AY796065	*L. broomii* strain 5399
EF612284	*L. licerasiae* serovar Varillal strain VAR010
Saprophytic ‘free-living’ *Leptospira*	
AY631876	*L. biflexa* serovar Patoc strain Patoc I
AY631878	*L. meyeri* serovar Ranarum strain Iowa City Frog
AY631879	*L. wolbachii* serovar Codice strain CDC
AY631882	*L.* genomosp. 5 serovar Saopaulo strain Sao Paulo
AY631888	*L.* genomosp. 4 serovar Hualin strain LT 11–33
AY631889	*L. meyeri* serovar Hardjo strain Went5
AY631892	*L. meyeri* serovar Semaranga strain Veldrat Samarang
AY631897	*L.* genomosp. 3 serovar Holland strain WaZ Holland
Leptospiral Sequences from Patient Urine Samples in this Study	
GU254499	PAD062 *L. interrogans*
GU254500	PAD254 *L. interrogans*
GU254501	PAD328 *L. interrogans*
GU254502	PAD115 *L. interrogans*
GU254503	PAD061 *L. interrogans*
GU254504	PAD117 *L. interrogans*
GU254505	PAD216 *L. fainei*
GU254506	PAD304 *L. fainei*
GU254507	PAD236 *L. broomi*
GU254508	PAD274 *L. licerasiae*

### Statistical Analysis

The proportion of the seropositivity rate (at any MAT titer) and distribution of the demographic variables were compared between the subjects with and without leptospiruria using the chi-square test and Mann-Whitney *U* tests using Stata v8 for Windows (StataCorp, College Station, Texas) with a significance level (*α*) of 0.05.

## Results

### Demographic Description of the Study Population

In the pre-study census and sampling period, 1320 people in 225 houses were identified in the Peruvian Amazon village of Padre Cocha near Iquitos. The study enrolled 354 participants of age ≥5 years from 175 households randomly picked from a census map. Of those 354, 40 participants were excluded since 19 presented with fever within 2 weeks of enrollment and 21 did not provide urine samples (patient enrollment diagrammed in Figure 1). The study included 314 participants with a median age of 27 (range 5—64). More were female than male (212 vs. 102); 63 (20%) were children younger than 15 years old (Table 2). Men (median  = 28.5 years, range (25%–75%) = (16.5 – 37) were on average slightly older than women (median  = 25, range  = 19–43) with borderline significance (*p* = 0.051).

**Table 2:** Characteristics of 314 asymptomatic participants from the Peruvian Amazon village of Padrecocha, Loreto Department, Peru.

	n = 314	(%)
Age median (min – max)	27 (5 – 64)	
Gender (M:F)	102∶212	
ELISA IgM positive	57/282	(20.2)
MAT positive (>1∶100)*	85/281	(30.2)
ELISA IgM and/or MAT positive	108/281	(38.4)
MAT high titer (>1∶800)*	13/281	(4.6)
ELISA IgM and/or MAT high titer (>1∶800)*	64/281	(22.8)
Fever within a year	171/314	(54.5)
Fever by malaria within a year	79/314	(25.2)

*Any serovar tested by MAT = Microscopic Agglutination Test. The ELISA incorporated serovars Icterohaemorrhagiae (RGA), Australis (Ballico), Bratislava (Jez Bratislava), Ballum (MUS127), Canicola (Hond Utretch IV), Cynopteri (3522 C), and Grippotyphosa (Moskva V).

### Prevalence of Leptospiral Seropositivity

Blood samples were available from 282 of 314 participants (89.8%). Of these, 97 were from males and 185 from females. Circulating anti-leptospiral antibodies were found, by either IgM ELISA or MAT or both, in 108 (38%) of the 281 subjects for whom serological data were available (Table 2). The most frequently observed serological reactivity (highest titer by MAT) was to serogroup Australis (34/281, 12.1%); with serogroups Djasiman (16/281, 5.7%), Icterohaemorrhagiae (13/281, 4.6%) and Cynopteri (12/281, 4.3%) also represented. Of the 108 seropositive samples, 64 had serological evidence of recent sub-clinical infection: seven had MAT titers (≥1∶800), 23 were IgM-positive but MAT-negative and 34 were IgM and MAT-positive, indicative of recent or current leptospiral infection. The proportion of MAT-positive (reflecting any previous exposure to *Leptospira*) and high MAT-titer (≥800, reflecting recent infection) persons was higher in men (40.6% and 9.4%, respectively) than women (24.9% (*p* = 0.006) and 2.2% (*p* = 0.006)). The difference stayed significant after adjusting for the age.

### Detection and Molecular Identification of Leptospiral Species from Urine Samples

**Figure 3:**
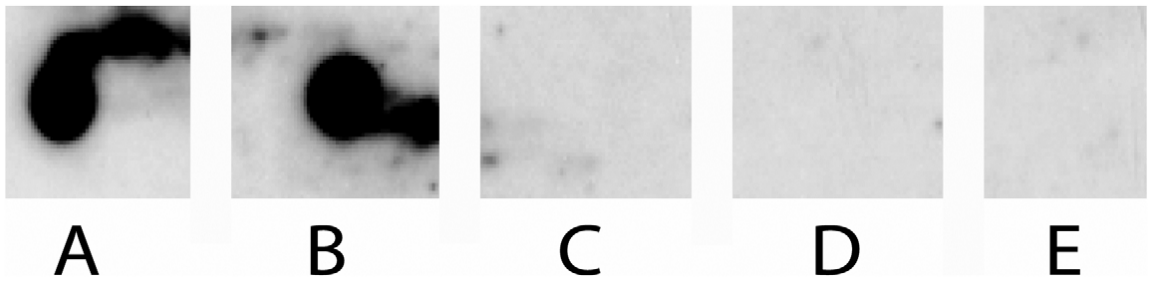
Dot blot hybridization confirming leptospiral DNA in patient urine samples. A =  *L. interrogans* ser. Icterohaemorrhagiae, B =  *L. licerasiae* ser. Var10, C =  *Atopobium vaginae*, D =  Negative urine, E =  Water.

The initial qPCR screening performed on-site in Iquitos detected 63 (20%) positive samples. Further evaluation of these samples with the nested PCR assay confirmed their positivity. Among these 63 PCR-positive samples, a newly designed dot-blot assay, designed to exclude false-positive samples containing only *Atopobium* DNA (Figure 3), identified 13/63 (21%) pCR-positive samples as true positives. We successfully cloned and sequenced the 16S rRNA gene from 10 of these dot-blot confirmed samples (Table 1); sequence data were not obtained from 3 samples. Species assignments were made by Bayesian phylogenetic analysis of the cloned 16S rRNA gene.

**Figure 4:**
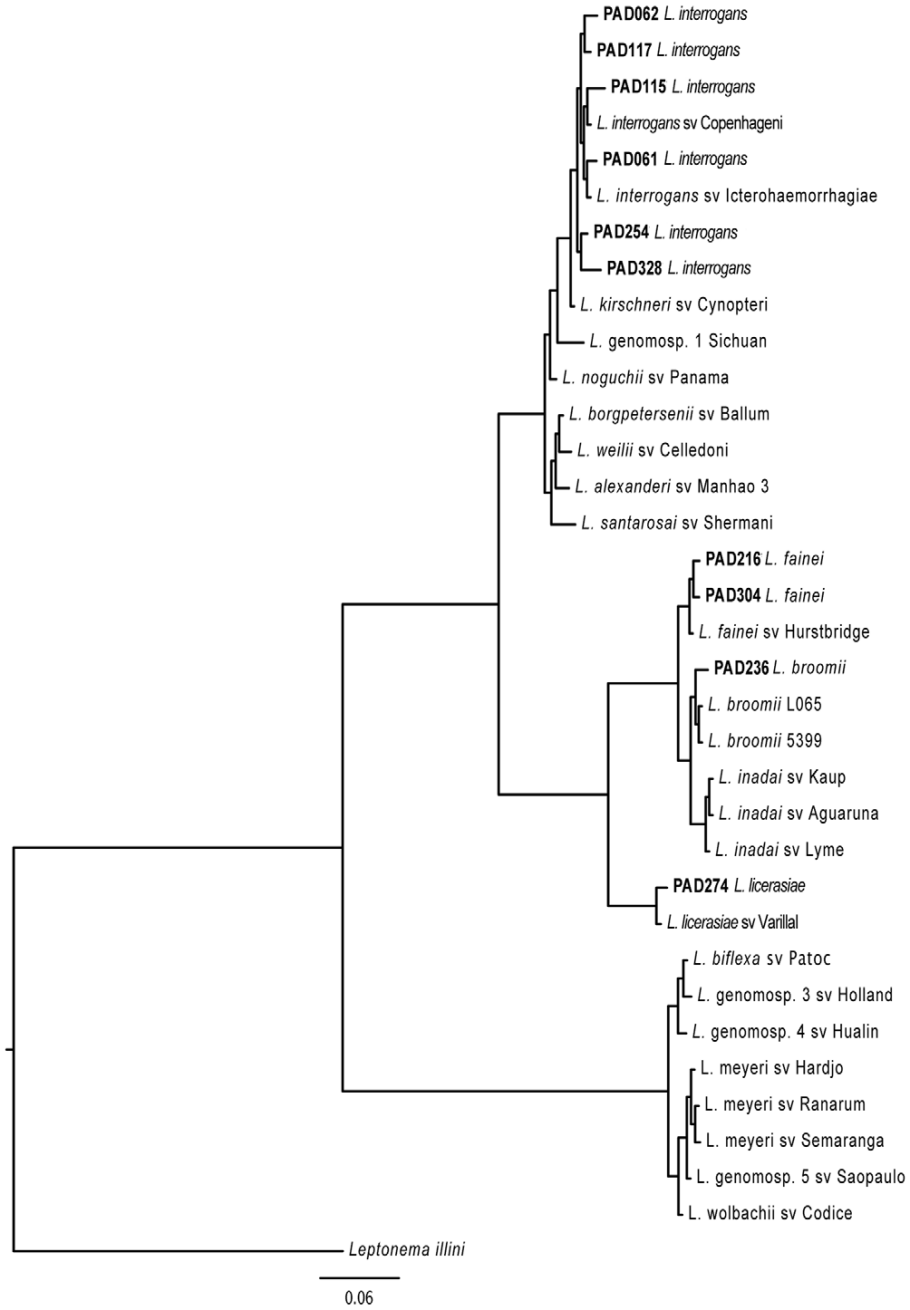
Phylogenetic relatedness of leptospiral 16 s rRNA gene sequences using a bayesian approach.

Analysis of these 10 dot-blot-confirmed urine samples showed that the 16S ribosomal RNA gene sequences clustered within both the pathogenic (n = 6) and intermediate clades of *Leptospira* (n = 4) (Figure 4). Although asymptomatic, one inhabitant (PAD304, Table 3) had serological evidence of acute infection (IgM-positive) indicating sub-clinical infection, and consequently excreted on average one hundred-fold more *Leptospira*/ml compared to IgM- and MAT-negative enrollees.

**Table 3:** Characteristics of *Leptospira* dot blot-positive subjects (N = 13).

ID	Gender	Age (years)	Fever ≤1 year	IgM	MAT	Species	Bacteria/mL urine
PAD115*	F	9	-	-	-	*L. interrogans*	1.37×10^2^
PAD216*	F	58	-	-	-	*L. fainei*	2.29×10^2^
PAD232*	F	44	-	-	-	NS	NS
PAD254*	F	29	-	-	-	*L. interrogans*	0.79×10^2^
PAD274*	F	31	-	-	-	*L. licerasiae*	0.75×10^2^
PAD328*	F	49	-	-	-	*L. interrogans*	2.26×10^2^
PAD062	F	50	-	NA	NA	*L. interrogans*	5.62×10^2^
PAD061	F	32	+	NA	NA	*L. interrogans*	2.27×10^2^
PAD117	F	52	+	-	-	*L. interrogans*	0.34×10^2^
PAD234	F	43	+	-	-	NS	NS
PAD236	F	46	+	-	-	*L. broomii*	0.32×10^2^
PAD285	F	40	+	-	-	NS	NS
PAD304	F	15	-	+	-	*L. fainei*	4.64×10^4^

*Subjects reported as long-term non-febrile (≥1 year) and IgM-negative.

NA = Serum sample not available, NS = Not able to be sequenced.

### Serological Results and Characteristics of the Participants with Leptospiral DNA in Urine

Serological results were available from 281 of the 314 participants including eight of the ten with *Leptospira* DNA positive urine (Table 3). Ten of 13 people with DNA-positive urine had negative results in both IgM and MAT.

All thirteen participants who had leptospiral DNA in their urine were women and the proportion (100%, 13/13) of the women was significantly higher compared to that in the leptospiral-DNA-negative group (66%, 199/301, *p* = 0.011). The median age of the DNA positive group (43 years, range (min – max) = 9−58) was older compared to the women in the negative group (median, 24; range, 5–60; years, *p* = 0.005). The difference stayed significant if the men were included in the negative group (median, 27 (5–64) years, *p* = 0.011). Univariate analysis did not show significant association between other epidemiological factors and leptospiral DNA positivity in urine (data not shown).

### Asymptomatic Urinary Shedding

Thirteen of the enrolled 314 asymptomatic inhabitants (4.1%) were confirmed to excrete *Leptospira* by detection of leptospiral DNA in their urine; of these, one participant may have had recent but sub-clinical leptospiral infection, based on an ELISA finding of IgM positive (Table 3). After clinical and epidemiological assessment, a group of asymptomatic participants was identified (n = 102, 32.5% of enrolled individuals) that had no evidence of recent infection (without febrile episodes in the previous year before enrollment and without anti-*Leptospira* IgM antibodies detected); we call them “long-term asymptomatic individuals.” Within this group, six (5.9%) excreted pathogenic and intermediate-pathogenic *Leptospira* (75–229 bacteria/mL of urine, Table 3).

## Discussion

This study has several important findings. First, asymptomatic individuals living in a region hyperendemic for leptospirosis had a high rate of seropositivity (at any level) for leptospiral infection (38% of 314 participants). Almost 60% of the seropositive individuals had evidence of recent sub-clinical infection, as indicated by MAT titer ≥1/800. Second, and of unique interest, a novel 16S rDNA hybridization assay used to screen urine samples for the presence of leptospiral DNA found that almost 5% of healthy people living in a rural Amazonian community were urinary shedders of *Leptospira* but did not have serological or clinical evidence of recent infection. Third, we found that both pathogenic and intermediately pathogenic *Leptospira* persistent infected the renal tubules of humans. Such observations have not been reported previously and are particularly notable because they demonstrate that inapparent leptospiral infection is common and frequently leads to shedding of organisms in urine. The long-term clinical significance of this finding remains to be determined.

The occurrence of leptospirosis, and indeed many infectious diseases, depends on several interacting variables. These include favorable environmental conditions, the density of local reservoir host populations, the type and frequency of exposure, exposure to infectious doses of the etiologic agent, the virulence of the infecting strain, and the lifestyle preferences and susceptibility of individuals within the exposed human population [21]. In the context of this zoonotic infection, the density of local animal reservoir populations is likely an important determinant of the extent to which the environment may become contaminated by leptospires through urine from chronically infected carriers. When environmental conditions are ideal and background contamination is prevalent, social practices that predispose to infection, and the virulence of local strains are significant factors that affect the incidence of the disease [1]. To date, there is no evidence that humans contribute to environmental contamination with *Leptospira*, but the data presented here do not rule out this possibility.

Exposure to *Leptospira* in this rural Amazonian study population was common (∼39% were serologically positive at any MAT titer) with many subjects having evidence of recent sub-clinical infection. However, the serological data presented here need to be interpreted with caution: in an endemic setting, a high individual MAT titer (≥1∶800) and/or IgM positivity are not reliable indicators of recent or current infection as antibodies may persist for prolonged periods [22]. The high background exposure rates and relative absence of severe disease in this hyper-endemic region do suggest that long-term urinary shedding may occur more frequently here than elsewhere, where natural immunity may not be as common.

It is generally accepted that humans can excrete leptospires from weeks to months after infection [23],[24]. However the data presented here indicate that humans may excrete *Leptospira* for periods exceeding a year; extending previous understandings of the carrier state. Ten asymptomatic individuals without clinical (no febrile episodes in more than a year) or serological evidence of recent exposure were found to be shedding either pathogenic or intermediately pathogenic *Leptospira* in their urine. Although these persons may have been recently sub-clinically infected and either failed to produce anti-leptospiral antibodies or all produced ‘false-negative’ serology, these explanations seem unlikely. It is more likely that they represent long-term renal asymptomatic shedders of *Leptospira*, regardless of whether patients were subclinically infected or had acute illness. However, a prospective study would be needed to assess this possibility. Nonetheless, prospective observational studies of such patients are required to confirm this hypothesis

Our data also suggest that women (especially mature women) are more likely to develop long-term renal carriage of *Leptospira* than are men; with a significant increase in incidence with age in women, possibly reflecting increased exposure with age or alternatively increased susceptibility. It is possible that the conclusion that women are more likely to be long-term asymptomatic urinary shedders than men may reflect a bias in the study, considering its relative underrepresentation of men. However, this observation may also reflect increased susceptibility of women to persistent leptospiral kidney infections; the reasons for this are unclear. However in our study population, the MAT titer was significantly lower in women than in men perhaps indicating that men are able to mount a more effective immune response than are women. Alternatively, men may have persistently higher antibody titers as a result of more frequent exposure due to work or recreational practices. Such possibilities require prospective study to address.

The long-term consequences of human renal infection by *Leptospira* need to be explored, in particularly the effect of persistent infection on renal function and electrolyte balance. Moreover, the nature of the infecting strains needs to be more carefully explored as some strains may be more likely than others to result in persistent renal infections in humans. Although we have identified the species of the infecting strains in the present study, other methods that are able to identify serovars, particularly isolation, will be more informative.

While humans are considered to be exclusively incidental hosts, animals can be maintenance and/or incidental hosts; maintenance hosts are defined as species in which infection is endemic, of low or no pathogenicity and (as a key factor) transmitted directly to the same species [25]–[28]. Although human-to-human transmission has been rarely documented, it is unlikely that asymptomatic infected individuals have an important role in disease maintenance and transmission [1],[11],[29],[30]. An increased risk of having leptospiral antibodies in households of leptospirosis index cases compared to controls in an epidemic setting has been shown recently [31], but this is most likely related to common environmental exposure risks or genetic susceptibilities rather than direct transmission. In light of these data, further studies should address the possibility that long-term urinary shedders may represent a source of *Leptospira* for their families and explore human-human transmission more carefully. Infection in carrier animals is usually acquired at an early age, and the prevalence of chronic excretion in the urine increases with age; we observed a similar trend in this population. Of note, none of the long-term urinary shedders had circulating anti-leptospiral antibodies; this is in accordance with early observations in *Leptospira*-carrier mammals, where chronic urinary carriage was associated with low seropositivity to urinary culture rates in asymptomatic well-established serovar-specific carriers [16]. Taken together, these observations make us speculate that in regions with high disease transmission, humans can develop some clinical and serological characteristics of asymptomatic urinary carriers, an attribute classically restricted to animals. Further longitudinal studies should address this possibility since the impact on disease transmission and in renal function of the affected individuals are unknown.

The study design had several limitations. First, we relied only on the recall of participants to define absence of fever in 1 year. Men were underrepresented; fewer men were recruited because of a lack of availability at the time of recruitment (most were away working). Thus, no leptospiruric males were detected. This observation suggests that we may have underestimated the overall number of asymptomatic shedders, as men have been typically associated with a higher risk of exposure due to work-related contact and behavioral practices. Another limitation was the initial non-efficient PCR screening strategy. The presence of other bacterial DNA hindered the identification of *Leptospira*-positive clones; we detected both *Leptospira* and *Atopobium* DNA in multiple samples, making the selection of colonies harboring the leptospiral 16S gene less efficient, in some instances, several hundred colonies had to be screened; we were unable to sequence the infecting strain in three enrollees due these technical limitations. Though unlikely, it is also possible that in these three instances the dot-blot gave false positive results. A third limitation of this study is that culture isolation of leptospires from urine was not attempted. Future work will be needed to further validate the molecular results presented here, and will use the PCR method to screen patients who then would have urine cultured for *Leptospira*. Nonetheless, the deployment of a valid molecular tool to detect leptospiruria represents a new approach to assessing chronic asymptomatic infections in humans without the need for obtaining isolates. Finally, because *L. licerasiae* serovar Varillal [7] had not been fully characterized nor its epidemiological implications known, this strain was not used as antigen in the MAT panel or ELISA used to study patient sera, nor are these sera available for retrospective analysis.

Few of the published L*eptospira*-specific PCR have been applied in clinical or field settings[32]. Furthermore, detection of bacterial DNA in urine is cumbersome because of the presence of PCR inhibitors and samples are often contaminated by multiple bacterial species whose DNA can interfere with the PCR assay [33].

Current understanding of host immune responses to *Leptospira* or the pathogenesis of leptospirosis remains limited. Naturally acquired immunity that protects against re-infection by *Leptospira* does occur and has been shown in animal models. It has been assumed that naturally acquired immunity is humorally-mediated particularly by antibodies against oligosaccharides of leptospiral LPS. Evidence also suggests that antibodies specific to *Leptospira* membrane-associated proteins may play a role in host defense [2],[34]. We have documented that in this hyperendemic area, in spite of the high levels of environmental exposure to *Leptospira* and high prevalence of seropositivity, the prevalence of severe disease is low [4],[7]. These observations suggest the possibility that protective immunity against severe disease from repeated infection may develop in areas with high leptospirosis transmission, especially if high frequency of infection leads to cross-serovar protection. Based on the finding that asymptomatic infection and urinary carriage are prevalent in this area where transmission is high and the prevalence of severe disease is low, we suggest that repeated exposure to *Leptospira* and asymptomatic infection could induce protective acquired immunity. Longitudinal studies are needed to test this hypothesis.

In conclusion, we have identified a long-term renal shedder group among persons asymptomatically infected with pathogenic and intermediately pathogenic *Leptospira*. The health implications of long-term renal colonization and whether antibiotic treatment of such patients is required remain to be determined.

## Supporting Information

Alternative Language Abstract S1 Japanese translation of the abstract by MS.
(0.03 MB DOC)

Alternative Language Abstract S2 Spanish translation of the abstract by CAG.
(0.03 MB DOC)

Checklist S1 STROBE checklist.
(0.09 MB DOC)
