# Supplementary material for: Asymptomatic Renal Colonization of Humans in the Peruvian Amazon by Leptospira
Source: PLoS Negl Trop Dis. 2010 Feb 23;4(2):e612. doi: 10.1371/journal.pntd.0000612 (PMC2826405; doi:10.1371/journal.pntd.0000612)
Supplement: Alternative Language Abstract S1 — Japanese translation of the abstract by MS. (0.03 MB DOC) [file pntd.0000612.s001.doc]

**要約**

レプトスピラが尿路系器官に保菌され潜在することは、保菌動物の特徴である。ヒトに感染した後にも、長期に渡って排菌されるという報告はいくつか散見される。我々は、ペルーアマゾン地域など感染率の高い状況では、ほかの哺乳類と同様にヒトでも無症候性のレプトスピラ尿を生じるのではないかという仮説を立てた。

**方法/主な結果**

無症候性のレプトスピラ尿を同定するため、疫学データ、血清学的所見、分子学的手法によるレプトスピラ16SｒRNAの検出を組み合わせた。無症状の研究参加者314名のうち、約3分の１に抗レプトスピラ抗体を認めた。参加者の59%（189/314）に血清学的に最近の感染を疑う所見（顕微鏡下凝集反応抗体価　[MAT] 1：800以上、またはELISA　IgM陽性。）を認めた。MAT陽性、あるいは高い抗体価を持つ者の割合は、女性に比べて男性に多かった（*p* = 0.006）。研究参加者の13名（4.1%）の尿中にレプトスピラのDNAを認め、そのうち10名の検体で16SｒRNAの遺伝子解析を行ったところ、病原性（n = 6）、中間型（n = 4）に系統分類されるレプトスピラを認めた。レプトスピラのDNAを尿中に認めた13名の全てが女性であった。DNA陽性であったグループの年齢の中央値は、DNA陰性であったグループに比べて高齢であった（*p* < 0.05）。血清学的、臨床的に最近の感染歴を認めないグループ（“長期間無症候者”、研究参加者の32.5% (102/341)）のうち、6名（5.9%）に、病原性あるいは中間型レプトスピラの排菌が認められた（75-229菌/ｍL尿中）。

**結論/意義**

レプトスピラの感染率の高い地域では、感染症状を認めないヒトの尿路系にレプトスピラが定着していることは稀でなく、これには血清学的、臨床的に最近の感染歴が疑われない人々も含まれていた。病原性、中間型レプトスピラのいずれもヒトの尿路系に定着する可能性が認められた。この所見の病理的意義は今後の課題であるが、基本的、生物学的意義のある結果が得られた。
