# Supplementary material for: Asymptomatic Renal Colonization of Humans in the Peruvian Amazon by Leptospira
Source: PLoS Negl Trop Dis. 2010 Feb 23;4(2):e612. doi: 10.1371/journal.pntd.0000612 (PMC2826405; doi:10.1371/journal.pntd.0000612)
Supplement: Alternative Language Abstract S2 — Spanish translation of the abstract by CAG. (0.03 MB DOC) [file pntd.0000612.s002.doc]

Resumen

Antecedentes

La condición de portador renal crónico y la excreción de leptospiras son características de animales huéspedes o portadores. Informes esporádicos indican que después de la infección, los seres humanos pueden excretar leptospiras por períodos prolongados. Nosotros hipotetizamos que, al igual que los mamíferos huéspedes portadores, los humanos pueden desarrollar leptospiuria asintomática en las zonas donde la transmisión de la enfermedad es alta, como la Amazonía peruana.

Metodología / Principales Hallazgos

Se utilizó una combinación de datos epidemiológicos, serología y detección molecular del gen 16S rARN de leptospira para identificar excretores urinarios asintomáticos de *Leptospira*. Aproximadamente un tercio de los 314 participantes asintomáticos presentaron anticuerpos contra *Leptospira* en sangre. Entre los participantes inscritos, 189/314 (59%) presentaron evidencia de infección reciente (prueba de aglutinación microscópica [MAT] ≥ 1:800 o ELISA IgM positivo o ambos). La proporción de personas con MAT positivo y MAT de título alto (≥ 1:800) fue mayor en hombres que en mujeres (*p* = 0,006). Entre éstas personas, 13/314 (4,1%) fueron positivas para ADN de Leptospira en muestras de orina. De éstos, el gen 16S rARN de 10 muestras pudo ser secuenciado. Las especies detectadas en orina se agruparon entre clados de especies patógenas (n = 6) y clados de especies de patogenicidad intermedia (n = 4). Todos los participantes con positividad para ADN de leptospira en orina (trece) fueron mujeres. La edad media del grupo ADN-positivo fue mayor en comparación con el grupo negativo (*p* ≤ 0,05). Un grupo de participantes asintomáticos ( "individuos asintomáticos de largo plazo", 102/341 (32,5%) de las personas inscritas), sin evidencia serológica de infección reciente fue identificado, dentro de este grupo, 6/102 (5,9%) excretó leptospiras patógenas y leptospiras de patogenicidad intermedia (75-229 bacterias/mL de orina).

Conclusiones / Significado

La colonización renal asintomática de leptospiras en una región de alta transmisión de la enfermedad es común, incluso entre las personas sin evidencia serológica o clínica de infección reciente. Leptospiras patógenas y de patogenicidad intermedia pueden persistir como colonización renal en los seres humanos. La importancia de este hallazgo queda por determinar, pero es de importancia biológica fundamental.
